# Faith, hope and love

**DOI:** 10.3325/cmj.2011.52.429

**Published:** 2011-06

**Authors:** Nika Matovac

Dear readers,

My name is Nika Matovac. I am a medical student and the daughter of Vesna Andrijević-Matovac. I would like to continue where my mum stopped, writing about how important it is to listen to the voice of patients. I dedicate this column to my mum, who raised me and was always there for me, despite her illness.

## Autumn 2001

That day I had a dancing lesson and my mum and grandma were supposed to pick me up, but only grandma came.

“Where is mum?” I asked her and she said “Don’t worry she is at the doctor’s and she will pick us up later at your friends place.” I was thrilled that I was going to play and dance with my friend and I had no idea what my mother was going trough that October evening visiting the doctor.

A few days later she got the results of the diagnostic examination and they were not good. I didn’t know what cancer was, but I knew it was not good and from that day on I was watching scared faces all around me.

Than it started – first surgery, then chemotherapy, then radiation, and all over again for several times. Although I was only 11, my mum and my dad explained to me what was going to happen, why my mum needed to remove her breast, why she was going to loose her hair, and why she might be sad from time to time. At first it was OK to deal with it because I was a kid and kids see everything with a happy ending.

Despite this constant battle with cancer, we had great moments together. It was winter and my mum, my grandma, and I decided to go for a walk in the snow. My mum was wearing a wig and was not able to put on a hat. When we came home, my dad opened the door and started laughing:

“Have you seen your wig?! Please look at yourself in the mirror, you look like British royal guard!”. When she looked herself in the mirror she also started laughing. The snow did not melt on her wig so she had this huge mass of snow on the top of the head. You just have to make fun of such a small things in this constant fight, otherwise it is too hard for everyone.

I wanted to share with you lots of funny moments to see that it was not only pain and tears, but all I can think of now is the last 3 years with my mum.

After 2001, we had a routine. I knew that she had to be given an infusion with her medicines every 3 weeks and that she had to make examinations from time to time to see if the cancer had spread. That was my mum’s life for the last 7 years but unfortunately the tumor was spreading.

My mum was a huge fighter and I would tell her: “Mum, you are so brave! I would never be able to fall asleep with the knowledge of having a cancer in me!”

I didn’t know how hard it was. I saw her crying sometimes but I couldn’t understand, no one who didn’t have the same illness could, and that’s why my mum started talking about how important it is to have real support, not only from your family and friends but from doctors and patients with the same problem. In the last 3 years, she did a great job about showing how important it is to hear the voice of patients and to spread it around!

During that time, I started to feel that I had a mum and a sister in one person… I will tell you why!

When I was 18 years old, I started going out with boys and my mum was very curious about every single detail connected with my love life. Then we went shopping and, because we were the same size, we would steal clothes from each other. On my prom night I was so happy to be with my parents and my mum enjoyed it, because she was constantly worried that she would not be able to see me graduating high school and be next to me when I start university.

## Christmas 2009

My mum just turned 49 (she was born on Christmas 1960) and she is doing OK with her cancer although they found an effusion in her left pleura. A year and a half ago she established, with 2 other patients, a patients’ association “EVERYTHING for HER!” and now she is trying to help other women with the same problem. Many times, my mum mentioned that the only thing she missed during her oncological treatment was psychological support and that’s why she wanted to do something about it.

Christmas time in my home was always special. I would decorate the Christmas tree, my dad would clean the house, and my mum would cook for Christmas Eve dinner – that was our annual routine. This Christmas Eve of 2009 was somehow special, because I started dating a boy from my university and my mum was very happy because she got an office for her association.

## January 2010

“Mum I have to tell you something!” I said.

“What is it!?” she asked.

“Well, I might have a boyfriend but this time… sort of a serious relationship!” – I replied blushing, because I was a bit embarrassed.

I really can’t describe mum’s expression but I can tell you that she was so happy to hear that, partly because she probably remembered her relationship with my dad and partly because she saw me turning into a woman.

From that day, she acted like a very nosey sister who wanted to know every single detail about my love and about how I felt in this relationship. She met my boyfriend and she liked him very much but also said several times: “Don’t do anything you don’t like and be yourself.”

## May 2010

Today the association “EVERYTHING for HER!” is finally opening the center for psychological support. This is the first center for psychological support in Croatia and even the president Dr Ivo Josipović is coming. My mum, as the host of this event, is very excited, and she looks amazing, and I know that no one would tell that she is 49 years old and that she had 3 surgeries and 5 chemotherapies behind her… today she is just shining.

Croatia got the first center for psychological support for women with breast cancer and their families and friends. I remember how crowded it was that day in the center and how many cameras were around my mum, but no one, not even me, didn’t know how she felt. During this opening she was dealing with a 1.8 L pleural effusion and shortness of breath. Cancer was destroying her and I couldn’t help her.

The next day, my mum and dad packed their bags and went to the “Klinikum Rechts an der Isar” in Munich. My mum spent almost 2 months there and had stereotactic radiation therapy of the left pleura. I was in Zagreb with my boyfriend studying for our histology and physiology exam. I wanted to be next to my mum but I knew that she would be happy if I passed all my exams before summer with good grades and that’s why I studied hard. News from Munich where rather good, but I would often ask: “Dear Lord, why is this happening to my mum? She is such a good person, she didn’t deserve this and I need her, I want her in my life!”

Now I can really thank my boyfriend because he gave me support and helped me concentrate on my studying.

## July 2010

My mum came back from Munich and the doctors are rather happy with the radiotherapy. I passed all my exams with great grades and now I am looking forward to the summer with my boyfriend in the USA.

## August 3, 2010

Today is a big day for me. I am going to the USA. My parents are here with me on the airport, kissing me, and giving me advice on how to behave in the unknown world and take care of myself.

“Enough kissing, mum, please!”, I said, “ I will come home soon!”, but mum being a mum worried about my journey.

I still remember how great that summer of 2010 was – I was with my boyfriend, exploring the whole new world, my mum was OK and recovering from her therapy, and I couldn’t even imagine what was going to happen in only 6 months. But, to be honest, I had some bad feeling.

## September 2010

I was back home in Croatia. My parents were waiting for me at the airport and I could see my mum’s face, she was so happy to see me, she couldn’t move the smile from her face. My dad was next to her holding my 14-year old maltese dog Pika. I really liked that picture of my little family, all together again.

## October 2010

My dog Pika is not doing very well and we are all scared that she might not survive. My mum needs to go on her usual control to Munich to see if everything is OK after radiotherapy.

Bad news again! Now I deal with them even harder than I did before. Pika is not recovering and she can’t walk and my mum got interstitial pneumonitis, a side effect of radiotherapy.

My little family was very attached to Pika but we couldn’t save her. Pika died on November 8, 2010. That day was very stressful especially for my mum because one of her friends with breast cancer also passed away.

Only 4 days after my dog Pika died, I had the first part of my pathology exam (they say that that’s one of the hardest exams in Medical School next to internal medicine), but I just couldn’t concentrate on studying. My mum was depressed because of her friend’s death and because of Pika and she was not feeling OK.

Somehow, thanks to my boyfriend Vanja and my parents, I found the strength to study and got an A. I dedicated the exam to Pika.

## November 15, 2010

From this day on, everything started to go downhill. My mum ended up in hospital with herpes zoster and pericardial effusion. Although I knew what pericardial effusion was and how hard that condition was, I still hoped that she would fight back like she had done for all 9 years. I would visit her every two days after my pathophysiology lectures, bringing her the Christmas spirit that was spreading through the city.

## Christmas Eve 2010

My mum is finally at home, after 2 months of “living” in the hospital. Again, there is a holy atmosphere in my home, my mum making dinner, dad cleaning the house, and me decorating the Christmas tree. This Christmas Eve is going to be a bit different, because we are going to spend it without my grandparents and my aunt and her husband, only the three of us, my mum, my dad, and me, and later my boyfriend will come. My mum is still feeling rather weak but I can see that she is happy.

## Christmas day 2010

“Mum, happy 50th birthday and Merry Christmas! I wish you all the best and above all health!”

## Late December 2010

I had a fight with my mum, nothing special, just regular mother and daughter fight, but I can feel my chest burning, burning in fear.

I was crying outside in the park and texted my mum:” Mum, I am scared, I don’t want to loose you. I want you next to me when I graduate, I want you to see my baby, I want you in my life, I don’t want you to die, mummy!”

My phone rang and I heard my mum telling me to come inside. We talked and talked and she finally said: “Nika, you must know that I am 50 years old now, that I have been dealing with this cancer almost 10 years, and I understand your fears but be aware that many people lost their mums even younger than you are now and that I am not that young any more and that you can be thankful that I am still here despite all I went through.”

My tears were just dropping down my face. I was speechless, but somehow peaceful.

## January 2011

My mum is again in the hospital at the cardiology department, with constrictive pericarditis. Doctors didn’t know whether that was a side effect of the radiation she had received in Germany or that was cancer attacking and squeezing her heart.

I didn’t loose hope. I was still hoping that my mum was going to be fine. I still hoped that she could deal with it.

## February 2011

My second pathology exam and also final pathology exam were close. My mum was still in hospital but now with severe anasarca, caused by constrictive pericarditis. I saw that she was not OK, but hope was still here. My dad and my boyfriend were trying to make a rather healthy atmosphere for studying and that’s why I had to find strength to study for my exam, and also because I knew that my mum would be very proud if I got a good grade in pathology. Despite my knowledge of how hard my mum’s condition was, I still hoped, hoped for a better tomorrow, tomorrow with a cure!

I passed my pathology exam, got an A… but 3 days later I lost one of the most important persons in my life, I lost my mum.

I dedicated the whole pathology exam to my mum, and I will always remember how great fighter she was and I will always remember that she provided me, despite her illness, more than a normal life, full of joy, laughter, and tears sometimes.

## May 15, 2011

Now while I am writing this column, I can’t stop thinking about how proud my mum would be if she could see how good this Center for psychological support is and how it helps other women and their families.

I can just say that I am, first of all, proud to be my mum’s daughter. I am also proud to be able to emphasize that not only doctors’ voice but also patients’ voices are important and that we can heal with words too… sometimes only one small word and gesture can make a difference and that’s why we all should stand next to our brave fighters, fighters for health and give them faith in a better tomorrow, hope until the very end, and love them always.

